# Endothelial Dysfunction and SARS-CoV-2 Infection: Association and Therapeutic Strategies

**DOI:** 10.3390/pathogens10050582

**Published:** 2021-05-11

**Authors:** Hai Deng, Ting-Xuan Tang, Deng Chen, Liang-Sheng Tang, Xiang-Ping Yang, Zhao-Hui Tang

**Affiliations:** 1 Division of Trauma & Surgical Critical Care, Department of Surgery, Tongji Hospital, Tongji Medical College, Huazhong University of Science and Technology, Wuhan 430030, China; d201981743@hust.edu.cn (H.D.); d202081897@hust.edu.cn (D.C.); m201976120@hust.edu.cn (L.-S.T.); 2 Class 1901, School of Medicine, Wuhan University of Science and Technology, Wuhan 430065, China; tangzh45@hust.edu.cn; 3 Department of Immunology, Tongji Medical College, Huazhong University of Science and Technology, Wuhan 430030, China; yangxp@hust.edu.cn

**Keywords:** COVID-19, SARS-CoV-2, endothelial cells, endothelial dysfunction, therapeutic strategies

## Abstract

Coronavirus disease 2019 (COVID-19), caused by severe acute respiratory syndrome-coronavirus 2 (SARS-CoV-2), has been recently considered a systemic disorder leading to the procoagulant state. Preliminary studies have shown that SARS-CoV-2 can infect endothelial cells, and extensive evidence of inflammation and endothelial dysfunction has been found in advanced COVID-19. Endothelial cells play a critical role in many physiological processes, such as controlling blood fluidity, leukocyte activation, adhesion, platelet adhesion and aggregation, and transmigration. Therefore, it is reasonable to think that endothelial dysfunction leads to vascular dysfunction, immune thrombosis, and inflammation associated with COVID-19. This article summarizes the association of endothelial dysfunction and SARS-CoV-2 infection and its therapeutic strategies.

## 1. Introduction

Since December 2019, a new β-coronavirus named severe acute respiratory syndrome coronavirus 2 (SARS-CoV-2) has led to an outbreak of respiratory disease around the world, which has posed a global challenge [1,2,3]. By 29 April 2021, it infected more than 148 million people worldwide and caused 3,128,962 deaths in over 200 countries (data compiled by Johns Hopkins University). Whereas most patients with COVID-19 show only mild symptoms, a proportion of patients develop severe complications within a short time after infection [2,4,5]. Both clinical studies and autopsy findings have shown evidence of vascular damage and thrombotic complications in multiple organs, such as acute ischemic or hemorrhagic stroke, myocardial injury, liver injury, acute kidney injury, as well as intestinal damage [6,7,8,9,10,11]. A number of studies have found that inflammatory processes, coagulation disorders, and microvascular thrombosis may exacerbate adult respiratory syndrome (ARDS) and extrapulmonary events in COVID-19 [12,13]. In addition, evidence suggests that the symptoms and signs of patients severely infected with COVID-19 are similar to the clinical phenotypes of endothelial dysfunction and have the same pathophysiological mechanism [6,14]. In particular, recent work by Chioh et al. found that COVID-19 patients, especially those with preexisting cardiovascular risk, may show signs of persistent endothelial dysfunction even after recovery from the infection [15].

Endothelial cells (ECs) play an important role in many physiologic processes. They control blood fluidity, leukocyte activation, adhesion, platelet aggregation and adhesion, and transmigration [3,16,17]. However, for most human viruses, ECs are the target of infection. Endothelial dysfunction is closely associated with organ dysfunction during virus infection [3]. The lung autopsy results of Ackermann et al. on seven patients with COVID-19 showed severe endothelial injury [18]. Increasing clinical evidence has shown that ECs participate in the pathogenesis of COVID-19 by reducing the integrity of the vascular wall, regulating clotting cascades, and mediating leukocyte infiltration [6,17,19,20]. 

To date, there are no specific drugs for COVID-19, and its pathogenesis remains largely unclear. Attention to the role of the endothelium in the pathophysiology of COVID-19 is of the utmost importance. In this article, we summarize the pathophysiology of SARS-CoV-2-induced endothelial dysfunction and potential therapeutic approaches for the treatment and prevention of COVID-19-related endothelial dysfunction.

## 2. The SARS-CoV-2 Virus

Coronavirus, order Nidovirales, family Coronaviridae, is composed of a helical capsid and a single-stranded RNA genome with a length of 27–32 kb [21,22,23,24]. This virus family includes four distinct genera, namely α, β, γ, and δ [22,25]. SARS-CoV-2, the causative pathogen of COVID-19, belongs to the β-genus and is considered a zoonotic pathogen that can infect a variety of species, especially birds and mammals [5,22,25]. Recently, researchers have completed the whole-genome sequencing of SARS-CoV-2 [22,26]. The SARS-CoV-2 genome contains five major open-read frames, which encode primary structural proteins, namely membrane (M), envelope (E), nucleocapsid (N), and spike (S) [25]. The M protein contains transmembrane domains that bind to the nucleocapsid and contribute to the shape of the virus [27]. The E protein is related to virus assembly and virus pathogenesis [27]. The N protein is mainly responsible for packing and encapsulating the genome into virions, and can antagonize silent RNA [24,26,27]. SARS-CoV-2 employs the S protein to recognize the target cells. The S protein comprises a trimer of glycoproteins, including two functional domains, the N-terminal region and the C-terminal region, named S1 and S2, respectively [24,25,26,28]. The S1 subunit is mainly responsible for recognition and binding the angiotensin-2 receptor (ACE2) on the cell surface [22,25,28,29]. The S2 subunit constitutes the stalk of the S protein, which plays a key role between the target cell and virus envelope, allowing the virus’s genetic material to enter the cell [22,25]. 

## 3. Infection of Endothelial Cells by SARS-CoV-2

Like other CoVs, SARS-CoV-2 relies on the ACE2 to enter the target cells, but has a higher affinity for ACE2 [5]. Studies showed that ACE2 is mainly concentrated on the surface of ECs and mucosal epithelial cells, such as the nasal and oral cavities, the lungs, and intestinal tract [30]. Although ACE2 is expressed on ECs of various organs, this does not mean SARS-CoV-2 invades all organs. There is no evidence that the virus invades the ECs of all organs so far. Coronavirus enters the cell mainly through endocytosis, which also depends on the recognition and binding of virus S protein to ACE2 and the initiation of S protein by host cell protease [31]. Therefore, coronavirus invasion of the cell depends on the expression of ACE2 and the involvement of transmembrane serine protease 2 (TMPRSS-2) or other proteases that cleave the virus S protein [32]. Previous studies showed that TMPRSS-2 is also expressed in human ECs [33]. Therefore, ECs expressing ACE2 and TMPRSS-2 are considered to be the target cells of SARS-CoV-2 [17,34,35]. 

The S protein mediates the virus to enter the host cells through a complex interaction with the ACE2 of the target cells. This interaction occurs in two key steps: first by attaching the viral S1 subunit to ACE2, and then by fusing the envelope of the virus to the membrane of the host cell [25,30,36,37]. Once the S protein binds to the ACE2 on the target cell, it causes some kind of conformational changes, making the S protein easily activated for membrane fusion [30]. These processes require the involvement of proteases including TMPRSS-2 and furin, which are essential for the membrane fusion of the virus with the cell [25,29,30,38]. They cleave the S protein between S1 and S2 subunits and rearrange its structure, allowing the envelope of the virus to fuse with the membrane of the target cell [28,29,30]. Once the virus enters the ECs, it begins to translate, replicate, and directly induce endothelial cell injury and apoptosis [17,25,39]. In severe COVID-19, the progressive infection of alveolar epithelial cells is accompanied by significant viral shedding, leading to necrosis and apoptosis [40,41].

## 4. Pathophysiology of Endothelial Dysfunction in COVID-19 Infection

### 4.1. Endothelial Dysfunction and Immune Responses

ECs in humans basically express both class I and II major histocompatibility complex (MHC) molecules, so they have important immune functions [3,42]. Costimulatory molecules and MHC molecules are usually necessary for T cell activation [17]. As ECs do not express costimulatory molecules CD80/CD86, ECs may function as semi-professional antigen-presenting cells (APCs) [17]. Thus, the number of T cells activated by ECs is far less than those activated by professional APCs. ECs can mediate the activation of Ag specific memory or effector CD4 and CD8 T lymphocytes, but cannot activate naïve lymphocytes [3]. It has been reported that vascular ECs could promote the clearance of cells infected by virus via presenting viral peptides to CD8+T cells [12,43]. Microvascular ECs have been reported to induce the trans-endothelial migration of memory or effector CD4 T lymphocytes [17,44]. In addition, cytokines such as IFN-γ can increase the expression of MHC class I or class II antigens in ECs [45]. Therefore, the endothelial damage and dysfunction mediated by COVID-19 would prevent ECs from activating lymphocytes, which may directly lead to an imbalance in the adaptive immune response [43]. 

Cytokines have been thought to play a critical role in immunopathology and immunity during the course of virus infection, but when released in excess or inappropriately, they can disrupt the normal protective function of the endothelium and enhance the pathological process [3,46]. IL-6 is produced by ECs in response to virus invasion and elevated in the circulation in the inflammatory state [17,47,48]. Clinical reports have shown that increased levels of IL-6 are related to COVID-19 pathogenesis [2,49]. IL-6 induces ECs to secrete pro-inflammatory/chemokines and activate C5a complement [3,50,51]. Evidence of elevated levels of C5a can be found in a number of clinical studies, including one that confirmed elevated levels of C5a in 39 patients with COVID-19 undergoing maintenance hemodialysis [12,52]. C5a mediates the activation and recruitment of leukocytes by binding to the receptor of C5a, thus promoting the degradation of vascular endothelial (VE)-cadherin and further leading to the destruction of the endothelial barrier [17,53,54]. Treatment of COVID-19 patients with anti-C5a antibodies has been shown to reduce systemic inflammation and increase lung oxygenation [12,55]. To date, the role of C5a in the pathophysiological process of COVID-19 remains unclear. 

ECs are speculated to be involved in the recruitment of leukocytes from the bloodstream to the inflammatory and infected sites [17,44,56]. In the process of SARS-CoV-2 infection, cytokines such as tumor necrosis factor-α (TNF-α) and interleukin-1 (IL-1) bind to the TNF receptor and IL-1 receptor on the surface of the ECs, which further initiates various kinase cascades and induces the expression of many kinds of adhesion molecules, such as VCAM-1, ICAM-1, P-selectin, and E-selectin [17,44]. VCAM-1 is a monocyte endothelial ligand independent of CD11/CD18, which not only recognizes α4β1 and α4β7 integrins in leukocytes, but also mediates monocytes’ recruitment from the bloodstream to sites of infection and injury [17]. ICAM-1, expressed on the surface of ECs and upregulated in lesions, supports leukocyte recruitment and adhesion and mediates the transition of lymphocytes and monocytes by binding with CC-chemokine ligand 2 (CCL2) [17,44]. P-selectin is mainly expressed on the endothelium and platelets, and plays an important role in the interaction between ECs and leukocytes, especially in regulating inflammatory pathways and preventing infections [17]. It has been found that the levels of adhesion molecules (VCAM-1, ICAM-1, P-selectin, and E-selectin) are significantly increased in patients severely infected with COVID-19 [17,57]. 

A clinical study reported that the levels of IL-6, IL-10, and TNF-α were significantly increased in COVID-19 [58]. In the context of SARS-CoV-2 infection, endothelial injury-mediated excessive inflammation is an important factor leading to lymphocyte depletion, as studies showed that IL-6 and TNF-α can induce lymphocyte deficiency [12]. Reduction in the number of CD4+ T lymphocytes may weaken the immune response to SARS-CoV-2 infection, and even aggravate excessive inflammation by down-regulating inflammatory mediators [12].Taken together, the above evidence indicates that the uncontrolled inflammatory response in severe infections with COVID-19 is closely related to ECs [59]. 

### 4.2. Endothelial Dysfunction and Thrombosis

ECs secrete various signals and mediators under normal physiological conditions that play a vital role in the prevention of pathological thrombosis [60]. The glycosaminoglycan on the endothelial surface binds to antithrombin III, resulting in the production of a potent thrombin inhibitor [61]. Thrombomodulin on the endothelial surface binds to thrombin, resulting in a decrease in activated protein C, which regulates coagulation activation by proteolytic cleavage of the cofactors VIIIa and Va [56,61]. ECs can also express a procoagulant protein inhibitor, which can prevent thrombosis induced by tissue factor [56,62]. In addition, ECs can synthesize and release a variety of relaxing factors, such as prostacyclin (PGI2) and nitric oxide (NO) [17,60,63]. NO can prevent the adhesion of leukocytes and platelets, the migration of inflammatory cells into the vessel wall, the proliferation of smooth muscle cells, and suppresses inflammation and apoptosis [64]. PGI2 and NO synergistically increase the content of cAMP in platelets, thereby preventing platelet aggregation and limiting the formation of thrombi [60]. In addition to PGI2 and NO, the lumen surface of ECs is covered by enzymes [60]. Ectonucleotidase hydrolyzes ATP and ADP, both of which are potent platelet-aggregating agents, into AMP and adenosine, thereby reducing platelet aggregation [60].

Whereas normal vascular lumen has anticoagulant, antithrombotic, and fibrinolytic functions, the anticoagulant and antithrombotic properties of the vascular lumen change when ECs are injured [56,60]. The tropism of SARS-CoV-2 for ACE-2 receptors expressed on ECs seems to result in ECs injury and apoptosis, leading to loss of the ability to maintain the physiological functions mentioned above [65]. Subsequently, the endothelial dysfunction leads to the procoagulant change in the vascular lumen and the formation of immune thrombosis. The autopsy studies of COVID-19-related deaths have reported clot formation, hyaline membrane in pulmonary arterioles, and diffuse alveolar injury [64,66]. 

### 4.3. Endothelial Dysfunction and Hypercoagulability

The endothelium secretes tissue plasminogen activator and has a glycocalyx layer, which prevents platelet binding or coagulation cascade triggering [47]. In addition, a variety of receptors are expressed on ECs, which have important antifibrinolytic, anti-platelet aggregation and anticoagulant activities. Thrombomodulin, a transmembrane receptor constitutively expressed on the surface of the ECs, can reduce the prothrombin activity of thrombin and favor activation of anticoagulant protein C after binding to the thrombin [67].

For patients with COVID-19, a hypercoagulable state is a critical condition [25,68,69]. Studies have shown that in this disease, elevated D-dimer levels are correlated with the worst outcomes and the hypercoagulable state is associated with higher mortality [8,10,25,70]. Compared with survivors, non-survivors had significantly longer prothrombin time, higher fibrin degradation product and D-dimer levels, and activated partial thromboplastin time [3,71]. This typical figure is considered to be the result of endothelial cell injury induced by SARS-CoV-2 infection. 

First, the endothelial injury results in the loss of the anticoagulant function of these cells and exposes underlying tissue factor to coagulation factors in the blood [60]. Second, destruction of endothelium integrity increases the exposure of the thrombogenic sub-endothelium to the vasculature, thus activating the intrinsic coagulation pathway [12,60]. Finally, endothelial dysfunction results in the massive release of von-Willebrand factor (VWF) from Weibel-Palade bodies, which has been reported in COVID-19 [12,72]. VWF is a glycoprotein secreted by ECs and platelets, which is stored in the Weibel-Palade body of ECs with Factor VIII [73]. Following endothelial dysfunction, this stored VWF is secreted, providing an effective bridge for platelet aggregation and thrombus assembly, which is conducive to the formation of organized clots [56].

In summary, SARSCoV-2 infection directly or indirectly causes endothelial dysfunction leading to a prothrombotic state through activating the internal and external coagulation pathways [12]. 

### 4.4. Endothelial Dysfunction and Vascular Permeability

ECs are the inner-most structure of all blood vessels and serve as the basic barrier between the interstitium and blood [60]. Controlling vascular permeability is one of the recognized functions of ECs in maintaining physiological homeostasis [17,60]. Under physiological conditions, endothelial gateways maintain vascular integrity by selectively regulating endothelial permeability [56,60]. VE-cadherin, known as the guardian of endothelial integrity, plays an important role in maintaining the integrity of the endothelial barrier [56,74]. In the context of SARS-CoV-2 infection, IL-1 stimulation can reduce VE-cadherin and thus affect endothelium integrity, resulting in capillary leakage [56]. In addition, the entry of SARS-CoV-2 through ACE2 induces a down-regulation of the expression of membrane-bound ACE2, which in turn may indirectly activate the kallikrein–kinin system (KKS), and eventually lead to the increase in vascular permeability [22,59,75,76]. Increased vascular permeability may contribute to extravasation and accumulation of fluids, proteins, and various inflammatory factors in the alveolar space and affect oxygenation function [56,76,77,78]. This finding directly links endothelial injury to capillary leakage and the exacerbation of ARDS as presented in advanced COVID-19 [56].

## 5. Therapeutic Strategies

To date, there are no specific antiviral drugs, and the treatment of COVID-19 patients is limited to symptomatic or palliative care. Given the above, therapies directly and indirectly aimed to prevent endothelial dysfunction and/or improve endothelial function may help to mitigate rapid disease progression and high mortality of COVID-19. Ongoing clinical trials are investigating treatments that target endothelial dysfunction associated with COVID-19. Therefore, we review the current therapies that prevent endothelial dysfunction and/or improve endothelial function in COVID-19.

### 5.1. Serine Protease Inhibitors

SARS-CoV-2 invasion of the cell mainly depends on the host TMPRSS-2, which plays an important role in the fusion of the S protein and the endothelial cell membrane. As such, inhibitors of synthetic serine protease, such as camostat mesylate, can negate the activating proteolytic processing of virus, and thus can theoretically prevent SARS-CoV-2 infection [64]. A recent study demonstrated that TMPRSS2, a protease inhibitor that can effectively prevent SARS-CoV-2 from invading cells, was tested in vitro and has been approved for clinical use [32]. Since ACE2 is required for viruses to invade cells, another method to prevent SARS-CoV-2 from invading cells is to use human recombinant soluble ACE2 [3,12]. Recombinant soluble ACE2 was proven to be a promising drug for the treatment of SARS-CoV-2 [12].

### 5.2. Renin-Angiotensin–Aldosterone System (RAAS) Inhibitors

Numerous studies have shown that both ACE inhibitors (ACEIs) and angiotensin receptor blockers (ARBs) can improve endothelial dysfunction [79]. ACE2 is not only a receptor for the virus invading the ECs, but also a vital part of RAAS [17]. As inhibitors of the RAAS upregulate ACE2 receptors [80], there were initial concerns about whether drugs such as ACEIs and ARBs would increase susceptibility to SARS-CoV-2 by upregulating the expression of ACE2 [81]. A number of clinical trials are underway to study the role of RAS inhibitors on COVID-19 (Clinical Trials NCT04311177, NCT04312009, NCT04335786, NCT04353596, NCT04364893, NCT04364984, NCT04367883, and NCT04394117). Evidence from a meta-analysis showed that the use of ACEI/ARB did not aggravate the severity of COVID-19 and can reduce the mortality of COVID-19 [82]. The available data indicate that the use of RAAS inhibitors did not increase the risk of SARS-CoV-2 infection or cause adverse outcomes [40,81,83,84,85]. On the contrary, due to an increase in the production of angiotensin 1–7 and the catabolism of angiotensin II, ACE2 upregulation may be more helpful than harmful for patients infected with SARS-CoV-2 [86,87]. Evidence from cardiology showed that RAAS inhibitors such as ACEIs and ARBs can reduce the risk of thrombus formation [88]. Thus, RAAS inhibitors have a positive effect on other antithrombotic treatments of COVID-19 [47]. 

### 5.3. Statins

Apart from lipid-lowering actions, statins can improve endothelial function via different mechanisms, including inhibition of NADPH oxidase, improved coupling and increased expression of nitric oxide synthase, and suppression of pro-inflammatory signal transcriptional and transduction pathways [40]. Previous studies have found that statins are beneficial for endothelial function in patients with rheumatoid arthritis or in patients with cardiovascular disease [89,90]. Some evidence suggests that statins are helpful in viral pneumonia (such as influenza) [91], indicating that they may be a promising category of drugs for preventing vascular damage and treating endothelial dysfunction in COVID-19. Evidence suggests that treatment with statins can reduce the production of inflammatory biomarkers and improve the prognosis of COVID-19 [40,56]. Safety concerns about statin therapy (liver damage, kidney damage, and myotoxicity) may be the reason for the reluctance to use statins as an adjunctive therapy for COVID-19 [92]. These conditions may be more common in patients severely infected with COVID-19 [92,93]. Therefore, further clinical practice should be considered to obtain reliable evidence of the role of statins in COVID-19.

### 5.4. Heparin

Heparin has anti-inflammatory effects, protecting the ECs by reducing histone toxicity and decreasing vascular leakage and lung edema [17]. In addition, heparin has an antiarrhythmic effect and can even oppose classical RAAS activation [47]. Anticoagulation with low-molecular-weight heparin (LMWH) can improve the prognosis of patients severely infected with COVID-19 [12]. A retrospective study of COVID-19 in 17 hospitals in Spain confirmed that heparin can reduce mortality after adjusting for age and sex [94]. Many studies have shown that heparin is beneficial to selected high-risk patients with COVID-19 [95]. Current expert recommendations suggest the use of unfractionated heparin or prophylactic-dose LMWH in all hospitalized COVID-19 patients without contraindications [21,96]. However, many findings have shown that thrombosis can occur in patients severely infected with COVID-19 despite treatment with LMWH at therapeutic doses [19,47]. The recommended dose of LMWH has not yet been determined, and the results of ongoing clinical trials are eagerly awaited. A number of clinical trials are underway to understand the role of LMWH in the treatment of patients with COVID-19 (Clinical Trials NCT04373707, NCT04393805, NCT04492254, NCT04542408, and NCT04584580). 

### 5.5. Corticosteroids

In addition to direct injury of ECs caused by viral infection, widespread release of inflammatory factors can also lead to ECs damage [72]. Given the important role of inflammatory factors in the pathophysiology of COVID-19, anti-inflammatory treatment is worthy of careful clinical evaluation. Corticosteroids are steroid hormones with strong anti-inflammatory properties [3]. They affect the function of various immune cells, including B lymphocytes, T lymphocytes, dendritic cells, and ECs [3,97]. A retrospective study of 201 patients with COVID-19 confirmed that methylprednisone treatment may be helpful for patients with ARDS [98]. Moreover, Group et al. showed that dexamethasone can significantly decrease 28 day mortality in patients severely infected with COVID-19 [99]. Dexamethasone has been authorized by the U.S. Food and Drug Administration (FDA) for emergency treatment of COVID-19 [12]. A number of clinical trials are underway to study the role of glucocorticoids in the treatment of COVID-19 (Clinical Trials NCT04343729, NCT04344288, NCT04344730, NCT04348305, and NCT04381936).

### 5.6. Other Agents

Other therapeutic considerations to improve endothelial dysfunction include: cytokine-directed therapies (against IL-1, IL-6, and interferon gamma), HMGB1-RAGE/TLR4 signaling inhibitors, antioxidant drugs, complement inhibitors, VEGFA/VEGFR2 signaling inhibitors, and pharmacological modulators [17]. 

## 6. Conclusions

There is growing evidence that endothelial dysfunction plays an important role in SARS-CoV-2 infection, suggesting that physiological functions and immunology of ECs should be paid more attention. Therapies aiming to prevent endothelial dysfunction and/or improve endothelial function in conjunction with specific antiviral administration may be particularly helpful to improve outcomes in COVID-19. 

## Data Availability

All datasets presented in this study are included in the article; further inquiries can be directed to the corresponding author.
